# Genetic testing for long QT syndrome and the category of cardiac ion channelopathies

**DOI:** 10.1371/4f9995f69e6c7

**Published:** 2012-05-03

**Authors:** Stephen M. Modell, David J. Bradley, Michael H. Lehmann

## Abstract

Cardiac ion channel mutational analysis is a category of genetic testing used in clinical practice for determining the status of long QT syndrome, short QT syndrome, catecholaminergic polymorphic ventricular tachycardia, and Brugada syndrome genes in blood, saliva, or tissue from patients and family members at risk for cardiac events such as syncope and sudden death. Such testing is most informative following careful phenotypic characterization. Individuals with ion channelopathies may benefit from prevention (avoidance of triggers and predisposing drugs) and treatment (e.g., beta blocker therapy, implantable cardioverter-defibrillator (ICD) placement) modalities.

## Clinical Scenario

In patients with fainting (especially occurring during physical exertion or emotional/auditory arousal), seizures, a history of aborted cardiac arrest, a family history of sudden death, or who have themselves succumbed to sudden cardiac death (SCD), cardiac ion channel mutation testing, typically in conjunction with electrocardiography, can provide important information. Performed with cardiac evaluation, genetic testing may be used to determine the status of long QT syndrome (LQTS), short QT syndrome (SQTS), catecholaminergic polymorphic ventricular tachycardia (CPVT), and Brugada syndrome (BrS) genes from blood, saliva, or tissue specimens, including postmortem samples^1^ ^2^ ^3^. While these conditions demonstrate characteristic electrocardiographic patterns (LQTS – a prolonged QT interval on resting ECG (part of the Schwartz-Moss diagnostic score)^4^; CPVT – ventricular ectopy^5^; BrS – ST segment elevation^2^), these findings may be absent or inconclusive on a single clinical test. Similarly, post-mortem samples may be accompanied by insufficient clinical data for diagnostic classification. Genetic testing has been used to exclude one condition over another and to provide definitive diagnosis in many such situations^1^ ^6^ ^7^. For the patient whose symptoms, ECG, and/or other adjunctive tests^5^ ^8^ have led to a clinical diagnosis, the test can identify pertinent risks and assist management. In addition, relatives of affected patients, who may have no symptoms but are capable of passing on an abnormal gene, can be identified through techniques like cascade screening^1^ ^3^ ^6^ ^9^. Therapies for patients displaying mutations include avoidance of physical triggers and aggravating drugs, placement on anti-arrhythmic drug or beta blocker therapy^10^ ^11^ ^12^ ^13^, use of mechanical devices (pacemakers, ICDs)^2^, and sympathectomy surgery^4^.

## Test Description

Cardiac ion channelopathies result from adverse alterations in genes that code for protein subunits of cardiac ion channels^1^. The literature differentiates these channelopathies in terms of their subtypes (e.g., for long QT syndrome, LQT1, LQT2, LQT3, LQT4, LQT5, LQT6, …, LQT13) and the name of the gene affected (KCNQ1 for LQT1; KCNH2 for LQT2; SCN5A for LQT3; ANK2 for LQT4; KCNE1 for LQT5; KCNE2 for LQT6, …, KCNJ5 for LQT13). Genetic testing is available through a number of commercial and university-based genetic diagnostics laboratories^1^ ^14^ ^15^ ^16^. Transgenomic (formerly PGx Health and Genaissance), GeneDx, and Correlagen all offer genetic assays allowing genotyping of LQTS, SQTS, CPVT, and BrS. For example, panels offered by Transgenomic^17^ and GeneDx^18^ test for 13 and 12 LQTS genes, respectively, KCNQ1, KCNH2, andSCN5A (LQT1-3) mutations being most frequent; 3 SQTS genes – mutations in the KCNH2, KCNQ1, andKCNJ2 genes; 4 (Transgenomic) and 2 (GeneDx) CPVT genes, RYR2 mutations being most frequent; and 7 BrS genes, SCN5A mutations being most frequent. The Masonic Medical Research Laboratory covers these conditions for research purposes, but specializes in acquired as opposed to congenital long QT syndrome^19^. Samples are collected in an EDTA-containing tube; the DNA is isolated from fresh whole blood. DNA amplification by polymerase chain reaction (PCR) is then used to generate templates for direct sequencing. DNA, frozen blood, saliva, and other tissue samples such as buccal specimens have recently been accommodated by some labs^17^ ^18^. Detailed results are provided for each test panel, typically with a description of known literature on any identified mutation and its likelihood to cause disease. Focused testing for a single mutation relevant to a patient’s family, rather than a panel of potentially involved genes, is available at a lower cost.

## Public Health Importance

The public health importance of these inherited arrhythmia syndromes is highlighted by their potential lethality, mostly due to ventricular tachyarrhythmias^20^. Long QT syndrome may be responsible for as many as 3,000 unexpected deaths in children and young adults annually in the United States^21^. Catecholaminergic polymorphic ventricular tachycardia carries a high mortality in untreated cases^5^ ^7^. The current state of technology, however, does not enable arrhythmia screening of broad, potentially at-risk populations – athletes, all children, all patients exposed to QT-prolonging drugs, or all patients with a history of syncope^6^ ^16^. Subpopulations of sudden death victims are of great interest. It is estimated that approximately 25-35% of autopsy-negative or unexplained sudden cardiac death in the young, and 10% of sudden infant death (SIDS) may be attributable to mutations in either LQTS or CPVT susceptibility genes^22^. Brugada syndrome may be responsible for 4-15% of unexpected sudden deaths, particularly in individuals with an apparently normal heart^23^ ^24^ ^25^.

**Table of Cardiac Ion Channelopathies:** 

	Long QT syndrome (LQTS) 17 26 27 28 75	Short QT syndrome (SQTS) 17 18 29	Catecholaminergic polymorphic ventricular tachycardia (CPVT) 5 17 30 31	Brugada syndrome (BrS) 17 32 33 34 35
Prevalence	1:5,000 to 1:2,000	Rare - fewer than 30 cases published	1:7,000 to 1:10,000	1:800 (Japan); 1:6,000 (U.S. & Europe) type 1 ECG pattern
Annual mortality rate	0.3% (LQT1) 0.6% (LQT2) 0.56% (LQT3)	Unidentified	3.1%	4% (pts. with type 1 ECG pattern)
Mean age of first event	14 ± 12 yrs.	40 ± 24 yrs.	15 ± 10 yrs.	42 ± 16 yrs. (pts. with type 1 ECG)
Diagnostic Yield - Genetic Testing	75-80%	15-20%	65-75%	11-40% (see Clinical Validity)

In the case of LQTS, the specific genotype has prognostic value^1^ ^6^. In studies ranging from 250 to 300,000 genotyped individuals through age 60 drawn from the International LQTS Registry, the LQT3 genotype has demonstrated a 5- to 8-fold higher risk for life-threatening events compared to the LQT1 and 2 genotypes^36^. The LQT2 genotype displays a risk intermediate between LQT1 and LQT3^36^, though the statistical strength and ordering of this relationship depends on age, sex, and source of the cohort^10^ ^37^. Subgroup analyses have more precisely defined the impact of QTc duration and genotype in children and adolescents with LQT1 and 2^38^.

## Published Reviews, Recommendations and Guidelines

Systematic evidence reviews

The Hayes Inc. Genetic Test Evaluation (GTE) Program has prepared three evaluations of ion channelopathies: long QT syndrome, CPVT, and Brugada syndrome. These reviews were based on studies of primary literature retrieved from PUBMED and Embase in the following date ranges: LQTS – January 1, 1996 to June 16, 2009; CPVT - January 1, 1996 to February 10, 2010; and Brugada syndrome - January 1, 1996 to August 3, 2010^39^. DNA Direct, Inc. has published two systematic reviews of LQTS genetic testing through its Genomic Medicine Institute. The reviews, which the company makes available with interactive tools and decision support, are based on studies of primary patient data published between 2001 and 2008^40^. BlueCross BlueShield Association and Kaiser Permanente published a systematic review of genetic testing for long QT syndrome through the BlueCross BlueShield Technology Evaluation Center (BCBS Tec)^41^. The review was based on studies of primary patient data appearing in Medline and PUBMED published between 1990 and October 2007. The Australia and New Zealand Horizon Scanning Network released a Horizon Scanning Report on genetic testing for congenital long QT syndrome based principally on nine peer-reviewed articles, and detailing safety (e.g., false negatives), effectiveness, clinical utility (cost-effectiveness), and ethical (consent and privacy, harms from testing, access) considerations^42^. Investigative teams have published comprehensive clinical-epidemiologic reviews of both long QT syndrome as a family and several of the other channelopathies under Human Genome Epidemiology (HuGE) reviews (e.g., long QT syndrome^21^) and GeneReviews (e.g., Brugada syndrome^43^).

Recommendations by independent groups

Two independent reviews have concluded that genetic testing has diagnostic value, including for the identification of asymptomatic heterozygotes, for all four syndromes; definitive prognostic value for LQTS and weak or contingent prognostic value (depending on the nature of the findings) for CPVT and BrS; and practical value in the determination of therapy for LQTS alone^1^ ^30^. One of the teams also published an “evidence-based” genetic testing scoring system to compare the relative clinical value of genetic testing over these conditions (LQTS > CPVT > BrS > SQTS)^44^. SQTS’s low score is associated with paucity of data, this condition having first been described in 2000^15^. Criteria on when to screen for each channelopathy, based on consensus documents and primary literature from 1995 onward, are laid out by Tzou and Gerstenfeld^15^. This review concluded that genetic testing can lead to genotype-specific therapy in the case of LQTS, and decisions about ICD placement for malignant arrhythmias associated with CPVT and SQTS^15^.

Guidelines by independent groups

A 2007 consensus report by the U.S. National Heart, Lung, and Blood Institute and the Office of Rare Diseases on gene mutations affecting ion channel function concluded that genetic testing for LQTS must be combined with clinical evaluation, and noted lack of clarity in the proportion of SQTS cases that might be explained by the corresponding KCNH2, KCNJ2, and KCNQ1 genes^45^. A 2011 Heart Rhythm Society (HRS) / European Heart Rhythm Association (EHRA) consensus statement further states that LQTS genetic testing is recommended for any asymptomatic patient with idiopathic (not attributable to QT prolonging disease states or conditions) QTc values > .48 s. (prepuberty) or > .50 s. (adult), and may be considered for QTc values >= .46 and .48, respectively^6^. (QTc = "heart rate-corrected QT interval," as per the Bazett formula^4^.) The Heart Rhythm UK Familial Sudden Death Syndromes Statement Development Group published in 2008 a position statement on genetic testing for sudden cardiac death syndromes based on a comprehensive review of English language publications, grading of the evidence, and secondary review of the evidence by an external committee^3^. The Group followed with a position statement on ICD placement for these conditions based on risk of SCD^46^. The first position statement and the more recent HRS/EHRA report recommend genetic testing for all patients with a firm diagnosis of congenital LQTS and those with clinical features of CPVT (due to its severity, despite an acknowledged lower clinical sensitivity), but that expert clinical and family history assessment are needed when genetic testing is undertaken for borderline LQTS cases and known or suspected cases of BrS. Practice guidelines from the American College of Cardiology / American Heart Association / European Society of Cardiology^47^ have noted an evolving role for genetic testing of LQTS in risk stratification and clinical decision making. Both independent reviews and professional society guidelines agree that genetic testing by itself is not recommended in making a diagnosis or prognosis for BrS, though it may be used to support clinical diagnosis, and early detection of at-risk relatives^6^ ^25^ ^48^. Several HRS / EHRA consensus statements clarify that genetic testing can be useful in patients clinically suspected of having BrS with a type 1 (“coved” ST segment elevation) ECG pattern, but that it is not indicated in the setting of an isolated type 2 (less specific, “saddleback” ST elevation) or 3 (either shape but less pronounced elevation) pattern^6^ ^20^ ^25^.

## Evidence Overview

Analytic Validity:

Commercially available channelopathy genetic testing (CGT) for LQTS, SQTS, CPVT, and BrS involves direct sequencing of protein-coding portions and flanking regions of targeted exons following PCR amplification. Sequencing is performed in both forward and reverse directions. Sequences are analyzed for heterozygous and homozygous variants using public reference sequences^17^ ^41^. For the FAMILION® test, variants detected in the initial analysis are confirmed by repeating the sequencing twice in the forward and three times in the backward direction. Transgenomic reports a CGT error rate of < 1% 17. GeneDx indicates a 98% “technical sensitivity” for assessment of each of the four conditions^18^. The John Welsh Cardiovascular Laboratory at Baylor College of Medicine makes available genetic diagnostic testing using DNA sequencing analysis for KCNJ2 (LQT7) and CAV3 (LQT9) mutations. This facility reports approximately 99% detection of the exons that are sequenced^49^. Failure to detect variants in the laboratory setting is attributed to refractoriness of the amplicon to analysis by direct DNA sequencing or real-time PCR detection, sample mishandling, sample tracking errors, errors in data analysis, and other gene specific issues (see Clinical Validity)^17^. The FAMILION® analytic specificity approaches 100% for “Class I” (deleterious or probably deleterious) mutations, and approximates 95% for “Class II” mutations (of uncertain clinical significance – possibly deleterious)^41^.

Clinical Validity:

The near perfect analytic sensitivities of these assays must be contrasted with the ability to detect genetic variants in the clinical setting. The yield for the first 2,500 consecutive unrelated cases referred by physicians for commercially available long QT syndrome genetic testing (low-, intermediate-, and high-pretest probability) was 36%; values in the literature range between 33 and 39%^50^. Tester et al. reported greater yields of 72 to 78% using the 5 major LQTS genes for patients with the highest clinical probability for LQTS (Schwartz-Moss score >= 4 4)^37^. Transgenomic reports a 75 to 80% yield for such patients based upon multiple data sets^17^ ^51^. False negatives may be explained by a number of factors, including the existence of private mutations^37^, the presence of non-targeted exons^17^ and relevant introns outside tested splice sites^16^, and the existence of large deletion and duplication mutations (generally on the scale of a whole exon or more)^7^ ^17^. LQTS is in the upper range among cardiac conditions in terms of number of affected genes (13) and allelic mutations (>800) while BrS lies in the midrange (8 genes and >400 allelic mutations). By comparison, current compendia of arrhythmogenic right ventricular dysplasia/cardiomyopathy list 9 affected genes and >400 allelic mutations, while Marfan syndrome has ~600 allelic mutations in the FBN1 fibrillin gene.

Authors have reported various mutational hotspots in the case of LQTS arising either independently or due to founder effects, which because of their severity may aid risk stratification^21^. Interpretation of results for probands and family members is complicated by variable penetrance in family members with the same genotype^52^, and the possibility of compound mutations, observed in 4 to 10% of mutation positive individuals^37^ ^50^ ^53^ ^54^. Estimated positive predictive values (EPV - percent of mutations found in definite cases that would cause the condition) for LQTS genetic testing based on an assessment of > 1300 unrelated index cases with Schwartz score >= 4 or QTc >= .48 s. are: 96% (95% C.I., 94-98) for *KCNQ1*; 93% (89-95) for *KCNH2*; and 63% (40-77) for *SCN5A* 55. Nonmissense mutations have an EPV > 99% regardless of location^55^. EPVs for missense mutations range from 0% in the interdomain linker of *SCN5A* to 100% in the transmembrane/linker/pore regions of *KCNH2*. Similar figures are lacking for the other channelopathies.

Several groups have investigated ECG genotyping (to be distinguished from use in initial diagnosis) through T-wave morphology as a means of reducing the cost of LQTS mutational genotyping^56^ ^57^ ^58^. Sensitivity is highly dependent on the gene being assessed, and varies between 92% (LQT2) and 47% (LQT3)^57^ ^58^. QTc interval alone lacks predictive value for genotyping^56^ ^57^.

For channelopathy genetic testing (CGT) of the other inherited arrhythmia syndromes, Transgenomic reports a clinical sensitivity of 65-75% (CPVT), 25-40% (BrS), and 15-20% (SQTS)^17^. GeneDx^18^ and the Correlagen CardioGeneScan®^59^ also test for these conditions. The GeneDx and Correlagen test information / FAQ sheets provide rough estimates of clinical sensitivity; figures from Correlagen are also reported with the assays. Some commercial laboratories only provide CPVT screening of a limited number of select exons encompassing critical *RYR2* regions, which can contribute to false negatives^30^. Disagreement exists on the impact of combining other variables with mutational results in order to increase clinical sensitivity; results vary by arrhythmia syndrome, genotype, and size of the study population^10^ ^27^ ^60^.

Mutational analysis of 27 *SCN5A* exons on cases from BrS databases at 9 international centers resulted in yields of 11-28%^35^ ^43^, lower than the 25-40% figure Transgenomic reports^17^ ^43,60^. The literature suggests that for BrS about a quarter of the patients or fewer carry an *SCN5A* mutation (commercial assays also include other lower frequency mutations)^1^ ^2^ ^25^. The low clinical sensitivity of genetic testing for Brugada syndrome, due to the low gene frequency for the most prevalent type of mutation (involving *SCN5A*), incompleteness of known allelic variants, and the role of an organic substrate in many cases, limits its diagnostic capability^15^ ^61^ ^62^. Genetic testing for BrS is more often used for confirmatory purposes^1^. Higher yields for the various channelopathies accrue in families with at least one recorded case of sudden unexpected death, permitting detection of potentially affected relatives^24^.

Clinical Utility:

Genetic analysis has been found useful in risk stratification of LQTS patients and detection of at-risk family members, though current knowledge of CPVT genetic variants does not in itself does not yield prognostic information^15^ ^22^ ^27^ ^30^ ^36^ ^41^ ^48^. “Intragenic risk stratification” based on mutation type and location, and cellular function, particularly for the LQT1 and 2 genotypes, has been an increasing part of large scale studies but is not yet in widespread practice^1^ ^6^ ^11^ ^41^ ^47^ ^63^. A meta-analysis of 30 Brugada syndrome prospective studies by Gehi et al.^64^ concluded family history of SCD and presence of an *SCN5A* mutation by themselves are insufficient to predict risk for cardiac events in BrS. Instead, debates about risk stratification for patients with this condition have taken place largely on the electrocardiographic front^65^. The presence of spontaneous ST-segment elevation, particularly with a type-1 pattern, in conjunction with a history of syncope remains the strongest predictor of BrS risk^2^.

Mutational information can serve as an adjunct to clinical and phenotypic assessment of genotype in therapeutic decisions for LQTS^30^ ^36^ ^48^, though this role is not without controversy^10^ ^12^ ^16^ ^41^ ^66^. Clear evidence exists for genotype-specific therapy in management of the LQT1, 2, and 3 genotypes, but it is less substantiated for the other LQTS genotypes due to their rarity^11^ ^12^ ^13^ ^36^ ^67^ ^68^. Disagreement exists on the use of intragenic, site-specific information to predict actual clinical phenotype or response to therapy for the LQTS genotypes^11^ ^37^. In analyzing retrospective data on 27 of 128 LQTS patients in the 3 to 13 year-old age range who received either pacemakers or ICDs as therapy, investigators in a 3-area study (Utah, British Columbia, and Arizona) found no association between device placement decisions and implementation of genetic testing. However, the authors also noted that all patients with *SCN5A* (LQT3) mutations had therapeutic devices, though only 1 of 30 with a *KCNQ1* (LQT1) mutation had one^69^. The use of genetic testing in therapeutic decision making for the other inherited arrhythmia syndromes is not yet substantiated but appears promising^16^ ^30^ ^48^ ^70^. ECG remains the principal tool for BrS diagnosis and therapeutic follow-up^2^ ^16^ ^25^.

Several teams have evaluated step-wise or tiered strategies also including phenotypic assessment to increase the efficiency of LQTS and CPVT genetic testing^1^ ^7^ ^53^ ^71^ ^72^. Targeted screening based on phenotypic information can lower the cost of more comprehensive LQTS genetic testing by ~60% 71. Phillips et al. found LQTS genetic testing more cost-effective than not testing for symptomatic index cases at an estimated cost of $2,500 per year of life saved ($50,000 per year of life saved is often used as a standard threshold)^73^ ^74^. Bai et al., in looking at 546 patients referred to a large consortium of LQTS research laboratories, found the highest yield (64%) and lowest cost ($8,418 per positive genotyping) for patients with ECG-confirmed LQTS, but diminishing yields and increasing costs for patients with borderline QTc intervals and those with normal intervals but a positive family history for SCD^60^. They recommended genetic testing be prioritized to those with a “conclusive diagnosis” of LQTS. Genotyping of individuals with a conclusive diagnosis of CPVT, and of patients with type 1 BrS ECG with atrioventricular block was also found to be cost-effective. Apart from this report, the host of cost-effectiveness analyses for Brugada syndrome deal with the issue of implantation of ICDs.

Perez et al. used a Markov model to assess the cost-effectiveness of different strategies for testing then treating an asymptomatic 10 year-old first degree relative of a patient with clinically evident LQTS^75^. They concluded that genetic testing is moderately expensive, at $67,400 per quality adjusted life year saved, but improves with higher clinical suspicion of the proband, number of relatives tested, and stronger family history of sudden death.

CGT is covered to different extents by insurance providers, ranging from denial to 100% coverage, with most covering at least 50 to 75% of the cost^22^. Some health plan policies explicitly limit genetic test reimbursement to LQTS while excluding the other channelopathies^76^.

Public Health Ethical, Legal, and Social (PHELSI) Considerations:

Genetic testing for cardiac channelopathies is laden with ethical issues, including the search for a balance between individual privacy vs. alerting at-risk family members, as well as psychosocial issues inherent in informing individuals of their risk^1^ ^77^ ^78^. A review by Wren provides general recommendations for genetic testing of children in families with a history of SCD, and offers ethical considerations in childhood genetic testing for Brugada syndrome^79^. Use of pacemakers and ICDs in children, while often done under situations of medical necessity, can involve trade-offs between benefit and adverse effects^45^ ^69^ ^80^. Screening for LQTS mutations in particular racial-ethnic groups deserves further ethical analysis^21^. Personalization of drug treatment through determination of individually specific genotype remains a hoped-for future direction in the field^1^ ^75^. Risk assessment for inherited heart arrhythmias is also becoming a part of direct-to-consumer genetic testing, an area subject to increasing attention by policy makers.

## Links

The commercial and university-based laboratories cited above are approved under the Clinical Laboratory Improvement Amendments (CLIA). Several, but not all, are accredited by the College of American Pathologists (CAP). These assays are developed and validated in-house, thus do not require FDA approval.

Relevant web sites:

- IRCCS Fondazione Salvatore Maugeri Molecular Cardiology Laboratories. Gene Connection for the Heart Inherited Arrhythmias Database. www.fsm.it/cardmoc. Up-to-date compendium of inherited arrhythmia syndrome (cardiac ion channelopathy, arrhythmogenic right ventricular cardiomyopathy, and others) mutations and polymorphisms, and condition synopses.
- Heart Rhythm Society. www.hrsonline.org. International society concerned with education and advocacy for cardiac arrhythmia professionals and patients. Web site describes and provides access to professional educational programs, clinical guidelines and consensus statements relating to diagnosis and management, and relevant legislation.
- Cardiac Arrhythmias Research and Education (CARE) Foundation. www.longqt.com. Advocacy and awareness-raising organization aimed at preventing sudden cardiac death due to acquired and heritable heart rhythm disorders. Web site reports professional educational resources, and details emerging advocacy issues, support groups, and genetic testing laboratories.
- Sudden Arrhythmia Death Syndromes Foundation. www.sads.org. Aimed at preventing sudden and unexpected cardiac death in children and young adults, the Foundation’s web site describes public awareness-raising activities, advocacy initiatives impacting patients and professionals, and patient and family support services.
- Drugs that Prolong the QT Interval and/or Induce Torsades de Pointes Ventricular Arrhythmia. www.qtdrugs.org. Web site provides a comprehensive list of arrhythmogenic drugs to be avoided by LQTS patients, lists drugs by risk group, provides consumer education tools, and describes the drug-induced arrhythmias case registry.
- Raymond Brugada Senior Foundation, www.brugada.org. Foundation web site provides a full description of Brugada syndrome, cites relevant literature and professional policies, and offers avenues for partnering in research and joining a support group.
- BrugadaDrugs.org. www.brugadadrugs.org. Web site provides a comprehensive list of arrhythmogenic drugs to be avoided by Brudaga syndrome patients, lists drugs by risk group, cites drugs diagnostic for Brs, and offers a patient letter listing drugs to be avoided.

## Competing Interests

The authors have declared that no competing interests exist.
